# Macrolide Resistance in the *Aerococcus urinae* Complex: Implications for Integrative and Conjugative Elements

**DOI:** 10.3390/antibiotics13050433

**Published:** 2024-05-10

**Authors:** Jyoti Lamichhane, Brian I. Choi, Natalie Stegman, Melline Fontes Noronha, Alan J. Wolfe

**Affiliations:** 1 Department of Microbiology and Immunology, Loyola University Chicago, Maywood, IL 60153, USAmfontesnoronha@luc.edu (M.F.N.); 2 Bioinformatics Program, Loyola University Chicago, Chicago, IL 60660, USA; nstegman@luc.edu

**Keywords:** *Aerococcus urinae* complex, uropathogen, macrolide, resistance, integrative and conjugative elements, ermA, horizontal gene transfer

## Abstract

The recognition of the *Aerococcus urinae* complex (AUC) as an emerging uropathogen has led to growing concerns due to a limited understanding of its disease spectrum and antibiotic resistance profiles. Here, we investigated the prevalence of macrolide resistance within urinary AUC isolates, shedding light on potential genetic mechanisms. Phenotypic testing revealed a high rate of macrolide resistance: 45%, among a total of 189 urinary AUC isolates. Genomic analysis identified integrative and conjugative elements (ICEs) as carriers of the macrolide resistance gene *ermA*, suggesting horizontal gene transfer as a mechanism of resistance. Furthermore, comparison with publicly available genomes of related pathogens revealed high ICE sequence homogeneity, highlighting the potential for cross-species dissemination of resistance determinants. Understanding mechanisms of resistance is crucial for developing effective surveillance strategies and improving antibiotic use. Furthermore, the findings underscore the importance of considering the broader ecological context of resistance dissemination, emphasizing the need for community-level surveillance to combat the spread of antibiotic resistance within the urinary microbiome.

## 1. Introduction

*Aerococcus urinae*, a Gram-positive coccus that grows in pairs and clusters, was first characterized from human urine in 1992 [1]. Originally described as a rare cause of human infection, a clear rise in diagnoses and case reports has been observed in the years since its first isolation [2,3,4,5]. The range of diseases associated with the bacterium includes malodorous urine, urinary tract infections, urgency urinary incontinence, urosepsis, soft tissue infections, and infective endocarditis, with antibiotic therapy as the primary treatment for infection [6,7,8,9,10,11,12,13]. As more and more strains have been studied, substantial phenotypic and genomic variation between strains has been observed, leading to the formation of subgroups [14,15]. It was from these subgroups that, just recently, new *Aerococcus* species were proposed, including *A. tenax*, *A. mictus*, and *A. loyolae* [16]. These three species, along with *A. urinae,* make up the newly described *A. urinae* complex (AUC) that more accurately describes the species diversity.

Similar to other emerging uropathogens, the AUC is poorly characterized and remains a blind spot in both disease and antibiotic resistance surveillance [17]. Worryingly, studies have been reporting antibiotic resistance, such as to fluoroquinolones, macrolides, and trimethoprim-sulfamethoxazole [18,19,20,21]. The current recommended empiric antimicrobial treatment for *A. urinae* urinary tract infection is the use of ampicillin, tetracycline, and nitrofurantoin [22]. However, published antibiograms do not regularly include AUC as part of surveillance testing, leaving rates of resistance unknown. These reports of resistance are concerning, especially given the global increase in uropathogenic resistance, particularly during the COVID-19 pandemic [23,24,25]. Resistant uropathogens are of particular concern as they may lead to further disease complications and recurrent urinary tract infections [26].

Within the human urinary microbiome, there is a lack of understanding as to how bacteria acquire antibiotic resistance, especially for emerging uropathogens [17]. The more commonly studied uropathogens, such as *Escherichia coli* or *Streptococcus agalactiae,* have been observed to engage in horizontal gene transfer with mobile genetic elements mediating the dissemination of antibiotic resistance genes (ARGs) [27,28]. As for AUC, it is unknown what genetic factors lead to observed resistances and whether horizontal gene transfer plays any role in acquired ARGs. However, the recent complete genome sequencing of the type strains of the new AUC species has enabled investigations into genetic determinants of resistance [29].

Thus, we sought to investigate any relationship between genotypic and phenotypic antimicrobial resistances of AUC isolates from urology and urogynecology patients at a United States quaternary medical center. We tested 189 AUC isolates for macrolide antibiotic susceptibility rates and found a pattern of persistent high resistance over a seven-year period between 2017 and 2023. Furthermore, we obtained evidence for bacterial horizontal gene transfer as the genetic basis for this pattern.

## 2. Results

### 2.1. Survey of Macrolide Susceptibility

A total of 158 AUC draft genomes from the National Center for Biotechnology Information (NCBI) were analyzed via comprehensive antibiotic resistance database (CARD) for ARGs. Assessing only perfect hits, 18 (11.4%) hits were predicted to encode macrolide resistance, 12 (7.6%) hits were predicted to encode glycopeptide resistance, and 9 (5.7%) hits were predicted to encode aminoglycoside resistance. We next sought to test whether strains within our local community hospital system demonstrate similar rates of macrolide resistance by characterizing the susceptibility of 189 AUC isolates.

**Figure 1:**
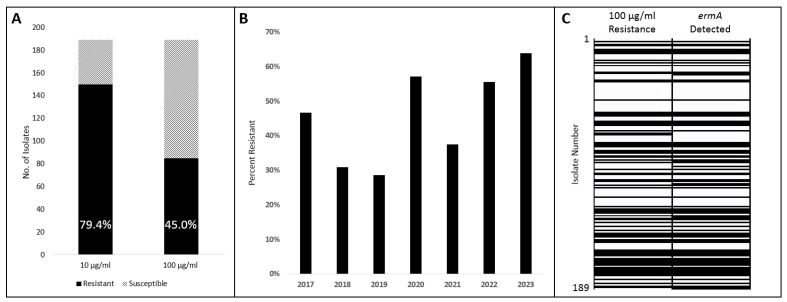
Azithromycin susceptibility and *ermA* presence among AUC isolates. (**A**) Susceptibility of AUC isolates out of a total of 189 tested at 10 μg/mL and 100 μg/mL. (**B**) Susceptibility of AUC isolates tested at 100 μg/mL compared by isolation year. (**C**) Concordance of phenotype (100 μg/mL) with genotype (*ermA* detection) of all 189 isolates. Isolates descending by isolation order with isolate number 1 earliest. Solid black indicates a positive result from either test.

Although the Clinical & Laboratory Standards Institute (CLSI) has published criteria for minimum inhibitory concentration (MIC) interpretation for AUC on several antimicrobial classes, it does not outline criteria for macrolides [30]. Considering that the already existing CLSI criteria were adapted from those of viridans group *Streptococcus* species, we at first adapted those criteria for azithromycin: susceptibility at ≤0.5 μg/mL and resistance at ≥2 μg/mL. A total of 150 (79.4%) isolates demonstrated resistance at the 2 μg/mL threshold; the same results were obtained at 10 μg/mL azithromycin. However, when we evaluated at our maximum concentration of 100 μg/mL azithromycin, 85 (45.0%) isolates demonstrated resistance (Figure 1A). Using this ≥100 μg/mL criterion, isolates were compared based on isolation year with resistances ranging between 29% and 64% (Figure 1B).

### 2.2. Survey of Macrolide Resistance Gene ermA

To evaluate a genetic basis for the high rate of macrolide resistance, a PCR test identifying the *ermA* gene was conducted on the same 189 AUC isolates. The PCR primers were designed based on the *ermA* sequence detected by the CARD analysis. *ermA* was detected in 89 (47.1%) isolates, which was similar to the 100 μg/mL Azithromycin resistance result of 85 (45.0%). Comparing these two experiments, 95.8% of isolates matched both the resistance phenotype and *ermA* genotype (Figure 1C). Of the 8 isolates that did not agree, 2 demonstrated a positive phenotype resistance but a negative *ermA* genotype, and 6 demonstrated a negative phenotype resistance but a positive *ermA* genotype (Supplementary Materials Table S1).

### 2.3. Macrolide Resistance Harbored within Mobile Genetic Elements

An initial analysis with VRprofile2 of the publicly available draft AUC genomes revealed that the macrolide resistance gene *ermA* was harbored within mobile genetic elements (Supplementary Materials Figure S1). These were identified to be integrative and conjugative elements (ICEs) capable of mobilizing DNA between bacterial chromosomes [31]. 

**Figure 2:**
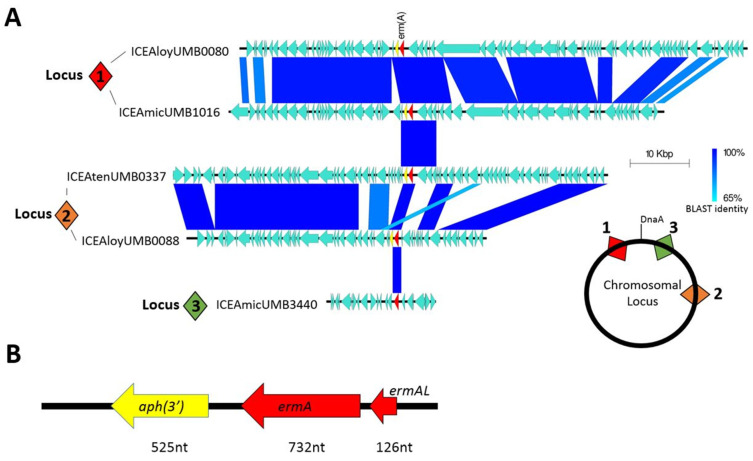
Macrolide resistance encoded in ICEs. (**A**) Homology comparison between different ICEs found in AUC isolates. Numbered square diamonds indicate syntenic loci for chromosomal insertion of each ICE. (**B**) Commonly inherited group of genes containing the *ermA* resistance gene.

To determine if this pattern of antibiotic resistance inheritance occurred in our isolates, we selected eight isolates that demonstrated resistance at the 100 μg/mL azithromycin threshold for whole genome sequencing. Although all eight genomes were found to actually possess the *ermA* resistance gene, the gene was not found in the same syntenic locus nor within the same ICE. Comparative genomic analysis revealed that ICEs containing the resistance gene integrated into AUC genomes at three distinct loci (Figure 2A). ICEs inserted at syntenic loci generally shared a high degree of nucleotide identity; however, very little nucleotide identity was observed between ICEs of non-syntenic loci. As one example, all ICEs found to insert within the *hsdM* type 1 restriction enzyme gene (locus 1) shared nearly 100% gene identity in ICE structural genes. But these ICEs only shared three open reading frames with ICEs inserted at locus 2 and only the *ermA* gene with ICEs at locus 3. Within the ICEs, the *ermA* gene was often inherited in combination with an upstream leader peptide and a downstream aminoglycoside 3′-phosphotransferase *APH* (*3*′) resistance gene (Figure 2B).

The majority of these ICEs belong to the Tn916 and Tn1806 ICE/dICE superfamily, which are commonly found in *Streptococcus* species. One ICE in particular, ICEAmicUMB3440, was found to be 97% identical to ICESag066, an ICE first documented in *Streptococcus agalactiae* (Supplementary Materials Figure S2) [28]. This ICE was the only one not found to contain the leader peptide-*ermA-APH*(*3*′) combination, instead only containing the *ermA* by itself within.

## 3. Discussion

This study sheds light on the prevalence of macrolide resistance in AUC isolates within a quaternary health system. In 2019, 260,000 global deaths were estimated to have been associated with antimicrobial resistance in urinary tract infections [32]. Although resistance to macrolides is not typically surveilled in organisms isolated from the urinary tract, azithromycin, primarily used for respiratory infections, can reach the bladder during excretion, exposing urinary tract bacteria to azithromycin [33,34]. Long-term use of macrolide antibiotics, such as azithromycin, has been linked to an increase in resistance, particularly in patients with conditions like COPD and cystic fibrosis [35]. Furthermore, a retrospective study found macrolide resistance to be the highest among common uropathogens isolated during the year 2021 [12]. In the current study, we found a persistently high rate of macrolide resistance in AUC isolates over a seven-year study period. Although this study period spanned the COVID-19 pandemic, it is undetermined whether the high rate of azithromycin administration during the COVID-19 pandemic influenced the macrolide resistance rates of uropathogens.

We identified the macrolide resistance gene *ermA* as a primary determinant of resistance within AUC isolates. Of our 189 isolates, 47.9% tested positive for the *ermA* gene by PCR, closely matching the 46.1% of isolates that exhibited resistance to high concentrations of macrolide. This gene was not inherited as a part of the AUC core genome but instead must have been introduced into the chromosome by means of horizontal gene transfer via ICEs. Interestingly, 1.8% of *ermA*-positive isolates remained susceptible to high azithromycin concentrations. This discrepancy could stem from the mobile genetic element itself being transcriptionally silent [36]. The emergence of macrolide resistance due to ICEs has been seen in other related uropathogens, such as *Streptococcus agalactiae* [17]. In fact, the same ICE characterized in *S. agalactiae*, ICESag066, was found to be within one of our AUC isolates. This observation implies that either the mobile genetic element can spread directly across species or may be received from a common donor within the bladder microbiome. Horizontal gene transfer of genetic content, not limited to ARGs, has been observed to occur in human-associated bacteria, and it may occur at an even higher rate in these microorganisms compared to environmental microorganisms [37,38]. Thus, emerging uropathogens not under routine resistance surveillance may pose a major health community threat by serving as reservoirs of resistance capable of spreading ARG-harboring ICEs to other species [39,40,41,42].

Contrary to the outdated belief of sterility, the urinary tract is made up of diverse polymicrobial communities [43,44]. The sharing of mobile genetic elements within this space poses a significant challenge to surveillance, highlighting the need to detect antibiotic resistance at a community level rather than cherry-picking select species [45]. Given the level of conservation of the *ermA* gene sequence between different ICEs and AUC isolates, our PCR test demonstrated a remarkable 95.8% concordance between the *ermA* genotype and resistance phenotype. As such, a potential future application may be to conduct whole-genome sequencing on bladder metagenomic DNA to evaluate urinary community resistance to macrolides. It would also be of interest to evaluate the movement of these ICEs between urinary bacteria, particularly when these mobile genetic elements move across species.

The implications of finding resistance in urinary bacteria towards an antibiotic class not primarily used in urinary infections provide valuable insight into the impact of off-target antimicrobial exposure. In the instance of macrolides, primarily used in respiratory infections, bladder bacteria are bystander organisms and are unintentionally exposed when the antibiotic is excreted from the body. Antibiotics are only partially broken down or absorbed in the body, with the rest excreted in urine or feces [46]. The concentration of many antibiotics, when excreted, is usually far lower than at the initial route of delivery, potentially priming off-target microorganisms towards resistance as a result of exposure to subinhibitory concentrations [47]. One study even found that subinhibitory antibiotic concentrations can promote horizontal gene transfer [48]. The emergence of off-target resistance is a major threat to patient health, especially when these off-target microorganisms serve as sources of future infections [49]. This speaks to the larger importance of proper antibiotic stewardship; however, a study of the US prescribing practices between 2017 and 2021 found that more than a quarter of prescribed antibiotics went towards conditions for which they are ineffective, providing unnecessary exposure to off-target microbiome bacteria [50].

This study underscores the critical role of mobile genetic elements carrying antibiotic-resistance genes and the emergence of antibiotic resistance. Emerging uropathogens like AUC remain greatly understudied, highlighting the urgency for comprehensive research. The clinical significance and resistance profile of these species are still unknown, emphasizing the need for in-depth exploration [17]. Our study has many features similar to how *S. agalactiae* has become recalcitrant to tetracycline therapy due to the dissemination of ICEs harboring resistance genes [51]. As such, a similar phenomenon may be occurring in other uropathogens, such as those belonging to the families *Aerococcaceae*, *Actinomycetaceae*, and *Bifidobacteriaceae*, for macrolide and other antibiotic resistances that have yet to be studied.

## 4. Materials and Methods

### 4.1. Survey of Publicly Available Genomes for ARGs

All publicly available AUC draft genomes were retrieved from NCBI (n = 158) and analyzed digitally at Loyola University Chicago with the CARD version 3.2.6 [52]. A complete list of genomes analyzed can be found in Supplementary Materials Table S2. Analysis of results was limited to perfect hits with default settings. Perfect hit refers to a perfect match to the curated reference sequences and mutations in the CARD database.

### 4.2. Study Isolates

The 189 AUC isolates included in this study had been previously recovered from male urology and female urogynecology patients at Loyola University Health Center between the years 2017 and 2023. These isolates were retrieved from a variety of sample sources, including voided urine, transurethral catheter urine, perineal swabs, vaginal swabs, periurethral swabs, kidney stones, and catheter tips as part of the Loyola University Chicago IRB-approved biorepository (LU 215192, 16 August 2021). Bacteria were isolated from urine and swab samples via the expanded quantitative urine culture (EQUC) method (44). A complete list of isolates can be found in Supplementary Materials Table S1.

Bacterial isolates were identified at Loyola University Chicago as AUC via matrix-assisted laser desorption/ionization time-of-flight mass spectrometry (MALDI-TOF MS) using the direct colony method. In this method, a sample of the bacterial colony is placed on a stainless steel target plate (Bruker Daltonics GmbH, Leipzig, Germany) before being treated with 70% formic acid. Upon drying, the α-cyano-4-hydrocinnamic acid matrix (Bruker Daltonics) is applied to the sample. The prepared sample plate is then analyzed by a MicroFlex LT mass spectrometer (Bruker Daltonics) with the MALDI Biotyper 3.0 software (Bruker Daltonics). *E. coli* DH5α was used as the quality control strain.

### 4.3. Phenotypic Screening of Macrolide Susceptibility

Macrolide susceptibility phenotypes of all 189 isolates were determined at Loyola University Chicago by broth microdilution technique in microtiter plates with 10-fold antibiotic dilutions. Overnight bacterial growth was inoculated in NYCIII media containing azithromycin (Sigma, Darmstadt, Germany) with a maximum antibiotic concentration of 100 μg/mL. The microtiter plates were then incubated within a BioTek Epoch 2 Microplate Spectrophotometer (Agilent Technologies, Wood Dale, IL, USA) in aerobic conditions with 5% supplemented CO_2_ at 37 °C with shaking at 200 rpm for 24 h. Optical density was measured every 30 min at 600 nm, and MIC was determined. For controls, PBS or bacteria was inoculated in NYCIII without antibiotics. All tests were conducted in duplicate.

### 4.4. DNA Extraction

To isolate genomic DNA from AUC isolates, 10 mL bacterial cultures were grown overnight in NYCIII media before being treated with lytic enzyme (lysozyme) at 37 °C for 60 min. Cells were lysed using the Wizard Genomic DNA Purification kit (Promega, Madison, WI, USA). The final DNA pellet was rehydrated in nuclease-free water overnight at 4 °C. Extracted bacterial DNA was stored at −20 °C until further processing. Genomic DNA was processed and stored at Loyola University Chicago.

### 4.5. PCR Survey

The presence of bacterial macrolide resistance gene *ermA* was detected by PCR and conducted at Loyola University Chicago. Forward primer: 5′-ACA TGA TAT TCC CTG TTT ACC CA-3′. Reverse primer: 5′-TGG AAA TGA GTC AAC GGG TG-3′. Each PCR reaction was carried out in a final volume of 50 μL consisting of molecular grade nuclease-free water (35 μL), 10× *Taq* buffer (5 μL) (Thermo, Waltham, MA, USA), 25 mM MgCl_2_ (3 μL), DNA template (3 μL), 10 mM dNTPs (1 μL), forward primer (1 μL), reverse primer (1 μL), and *Taq* polymerase (1 μL) (Thermo). The PCR reactions were performed in a SimpliAmp thermocycler (Applied Biosystems, Waltham, MA, USA). Thermocycling conditions were as follows: initial denaturation at 94 °C (10 min), 30 cycles of 94 °C (30 s) + 55 °C (1 min) + 72 °C (1 min), and final extension at 72 °C (7 min). PCR products were analyzed by separation on 1.0% agarose gels. A PCR reaction without template DNA was used as a negative control.

### 4.6. Genome Sequencing

Complete genome sequences of eight AUC isolates demonstrating resistance phenotypes were assembled by combining short-read and long-read sequences. DNA extraction and sequencing of isolates was performed by SeqCenter (Pittsburgh, PA, USA). For short reads, the Qiagen UltraClean Microbial Kit (Qiagen, Hilden, Germany) for extraction and the Nextera DNA Flex Library Prep kit (Illumina, San Diego, CA, USA) for library preparation were used before being run on the Illumina MiSeq or NovaSeq platform. For long reads, the Zymo DNA Miniprep kit (Zymo Research, Irvine, CA, USA) for extraction and Oxford Nanopore Technology Ligation Sequencing kit V14 (Oxford, UK) for library preparation were used before being run on the MiniION platform. Hybrid assembly was performed by combining filtered short- and long-sequence reads using SPAdes v3.15.4 [53], Flye v2.9 [54], and/or Canu v1.5 [55]. Genomes were polished with Pilon v1.24 [56], validated with QUAST v5.2.0 [57], and circularized using Circlator v1.5.5 [58]. Open reading frames were annotated via PGAP v6.6 [59]. Complete genomes were deposited and are publicly available at BioProject PRJNA316969 (Supplementary Materials Table S3).

### 4.7. Integrative and Conjugative Element Analysis

The identification and classification of ICEs were conducted by the same method used for the identification of *Streptococcus* species [60,61]. Briefly, genomes were queried via BLAST comparison against reference ICE proteins (e.g., integrases, transposases, Type IV secretion system proteins, and relaxases). Putative ICEs were delineated by syntenic comparison. The resistance gene *ermA* within ICEs was detected with VRprofile2 [62]. The homology of ICEs and *ermA* was visualized using EasyFig version 2.2.5 [63].

## 5. Conclusions

In this study, we characterized a concerning rate of macrolide resistance among AUC uropathogen isolates within a United States quaternary medical center. The identification of the *ermA* gene as a primary determinant of macrolide resistance highlights the role of horizontal gene transfer via mobile genetic elements, particularly ICEs, in disseminating resistance within bacterial populations. Our findings suggest that emerging uropathogens, such as AUC, may serve as reservoirs of resistance genes capable of receiving resistance determinants from other species within the urinary microbiome. This underscores the importance of understanding the dynamics of antibiotic resistance transmission within polymicrobial communities in the urinary tract.

## Data Availability

All complete genomes in the study have been deposited in NCBI. Links to the genomes can be found in the Supplementary Materials Table S2.

## Supplementary Materials

The following supporting information can be downloaded at https://www.mdpi.com/article/10.3390/antibiotics13050433/s1. Table S1. List of Isolates. Table S2. List of Analyzed Genomes. Table S3. NCBI Deposit of Complete Genomes. Figure S1. VRprofile2 output of ICEAloyUMB0088. Figure S2. Nucleotide homology of AUC ICEAmicUMB3440 and *Streptococcus agalactiae* ICESag066.

## References

[1] Aguirre M., Collins M.D. (1992). Phylogenetic Analysis of Some *Aerococcus*-like Organisms from Urinary Tract Infections: Description of *Aerococcus urinae* sp. nov. J. Gen. Microbiol..

[2] Senneby E., Petersson A.C., Rasmussen M. (2015). Epidemiology and Antibiotic Susceptibility of Aerococci in Urinary Cultures. Diagn. Microbiol. Infect. Dis..

[3] Narayanasamy S., King K., Dennison A., Spelman D.W., Aung A.K. (2017). Clinical Characteristics and Laboratory Identification of *Aerococcus* Infections: An Australian Tertiary Centre Perspective. Int. J. Microbiol..

[4] Sihvonen R., Turunen M., Lehtola L., Pakarinen L., Grönroos J.O., Rantakokko-Jalava K., Pätäri-Sampo A. (2022). Clinical and Microbiological Characterization of *Aerococcus urinae* Bacteraemias at Helsinki Metropolitan Area, Finland. Eur. J. Clin. Microbiol. Infect. Dis..

[5] Maezawa Y., Nagasaki K. (2024). Aerococcus urinae: An Emerging, Gram-Positive Pathogen Causing Urinary Tract Infection. Am. J. Med..

[6] Skalidis T., Papaparaskevas J., Konstantinou D., Kapolou E., Falagas M.E., Legakis N. (2017). *Aerococcus urinae*, a Cause of Cystitis with Malodorous Urine in a Child: Clinical and Microbiological Challenges. JMM Case Rep..

[7] Rasmussen M. (2016). *Aerococcus*: An Increasingly Acknowledged Human Pathogen. Clin. Microbiol. Infect..

[8] Pearce M.M., Hilt E.E., Rosenfeld A.B., Zilliox M.J., Thomas-White K., Fok C., Kliethermes S., Schreckenberger P.C., Brubaker L., Gai X. (2014). The Female Urinary Microbiome: A Comparison of Women with and without Urgency Urinary Incontinence. mBio.

[9] Sturm P.D., Van Eijk J., Veltman S., Meuleman E., Schülin T. (2006). Urosepsis with *Actinobaculum schaalii* and *Aerococcus urinae*. J. Clin. Microbiol..

[10] Zhang Q., Kwoh C., Attorri S., Clarridge J.E. (2000). 3rd *Aerococcus urinae* in Urinary Tract Infections. J. Clin. Microbiol..

[11] Ahmed Y., Bardia N., Judge C., Ahmad S., Malozzi C., Calderon E. (2021). *Aerococcus urinae*: A Rare Cause of Endocarditis Presenting With Acute Stroke. J. Med. Cases.

[12] Forsvall A., Wagenius M., Rasmussen M. (2019). Perigenital Necrotizing Soft Tissue Infection Caused by *Aerococcus urinae*. IDCases.

[13] Tai D.B.G., Go J.R., Fida M., Saleh O.A. (2021). Management and Treatment of *Aerococcus* Bacteremia and Endocarditis. Int. J. Infect. Dis..

[14] Christensen J.J., Kilian M., Fussing V., Andresen K., Blom J., Korner B., Steigerwalt A.G. (2005). *Aerococcus urinae*: Polyphasic Characterization of the Species. APMIS.

[15] Carkaci D., Højholt K., Nielsen X.C., Dargis R., Rasmussen S., Skovgaard O., Fuursted K., Andersen P.S., Stegger M., Christensen J.J. (2017). Genomic Characterization, Phylogenetic Analysis, and Identification of Virulence Factors in *Aerococcus sanguinicola* and *Aerococcus urinae* Strains Isolated from Infection Episodes. Microb. Pathog..

[16] Choi B.I., Ene A., Du J., Johnson G., Putonti C., Schouw C.H., Dargis R., Senneby E., Christensen J.J., Wolfe A.J. (2023). Taxonomic Considerations on *Aerococcus urinae* with Proposal of Subdivision into *Aerococcus urinae*, *Aerococcus tenax* sp. nov., *Aerococcus mictus* Sp. Nov., and *Aerococcus loyolae* sp. nov. Int. J. Syst. Evol. Microbiol..

[17] Moreland R.B., Choi B., Geaman W., Álvarez C.J., Hochstedler-Kramer B.R., John J., Kaindl J., Kesav N., Lamichhane J., Lucio L. (2023). Beyond the Usual Suspects: Emerging Uropathogens in the Microbiome Age. Front. Urol..

[18] Ahmadzada A., Fuchs F., Hamprecht A. (2023). Susceptibility of *Aerococcus urinae* and *Aerococcus sanguinicola* to Standard Antibiotics and to Nitroxoline. Microbiol. Spectr..

[19] Krishnan A., Nadeau L. (2019). 1468. Determination of Antibiotic Susceptibilities in *Aerococcus urinae* Urinary Isolates. Open Forum Infect. Dis..

[20] Humphries R.M., Hindler J.A. (2014). In Vitro Antimicrobial Susceptibility of *Aerococcus urinae*. J. Clin. Microbiol..

[21] Humphries R.M., Lee C., Hindler J.A. (2011). *Aerococcus urinae* and Trimethoprim-Sulfamethoxazole. J. Clin. Microbiol..

[22] Saad A., Hailes J., Jacobs M.R., Navas M.E. (2023). Antimicrobial Susceptibility Profile of *Aerococcus urinae*: Recommendations for Empirical Therapy. Infect. Dis. Clin. Pract..

[23] CDC (2022). COVID-19: U.S. Impact on Antimicrobial Resistance, Special Report 2022.

[24] Abdel Gawad A.M., Ashry W.M.O., El-Ghannam S., Hussein M., Yousef A. (2023). Antibiotic Resistance Profile of Common Uropathogens during COVID-19 Pandemic: Hospital Based Epidemiologic Study. BMC Microbiol..

[25] Mareș C., Petca R.-C., Petca A., Popescu R.-I., Jinga V. (2022). Does the COVID Pandemic Modify the Antibiotic Resistance of Uropathogens in Female Patients? A New Storm?. Antibiotics.

[26] Thänert R., Reske K.A., Hink T., Wallace M.A., Wang B., Schwartz D.J., Seiler S., Cass C., Burnham C.-A.D., Dubberke E.R. (2019). Comparative Genomics of Antibiotic-Resistant Uropathogens Implicates Three Routes for Recurrence of Urinary Tract Infections. mBio.

[27] Harris M., Fasolino T., Ivankovic D., Davis N.J., Brownlee N. (2023). Genetic Factors That Contribute to Antibiotic Resistance through Intrinsic and Acquired Bacterial Genes in Urinary Tract Infections. Microorganisms.

[28] Khan U.B., Portal E.A.R., Sands K., Lo S., Chalker V.J., Elita J., Spiller O.B. (2023). Genomic Analysis Reveals New Integrative Conjugal Elements and Transposons in GBS Conferring Antimicrobial Resistance. Antibiotics.

[29] Choi B.I., Fontes Noronha M., Kaindl J., Wolfe A.J. (2024). Complete Genome Sequences of *Aerococcus loyolae* ATCC TSD-300^T^, *Aerococcus mictus* ATCC TSD-301^T^, and *Aerococcus tenax* ATCC TSD-302^T^. Microbiol. Resour. Announc..

[30] Clinical and Laboratory Standards Institute (2016). Methods for Antimicrobial Dilution and Disk Susceptibility Testing of Infrequently Isolated or Fastidious Bacteria, M45.

[31] Johnson C.M., Grossman A.D. (2015). Integrative and Conjugative Elements (ICEs): What They Do and How They Work. Annu. Rev. Genet..

[32] Li X., Fan H., Zi H., Hu H., Li B., Huang J., Luo P., Zeng X. (2022). Global and Regional Burden of Bacterial Antimicrobial Resistance in Urinary Tract Infections in 2019. J. Clin. Med..

[33] Wildfeuer A., Laufen H., Leitold M., Zimmermann T. (1993). Comparison of the pharmacokinetics of three-day and five-day regimens of azithromycin in plasma and urine. J. Antimicrob. Chemother..

[34] Kim M., Welch T. (2014). Update on Azithromycin and Cardiac Side Effects. Southwest Respir. Crit. Care Chron..

[35] Gallacher D.J., Zhang L., Aboklaish A.F., Mitchell E., Wach R., Marchesi J.R., Kotecha S. (2024). Baseline Azithromycin Resistance in the Gut Microbiota of Preterm Born Infants. Pediatr. Res..

[36] Lipszyc A., Szuplewska M., Bartosik D. (2022). How Do Transposable Elements Activate Expression of Transcriptionally Silent Antibiotic Resistance Genes?. Int. J. Mol. Sci..

[37] Jeong H., Arif B., Caetano-Anollés G., Kim K.M., Nasir A. (2019). Horizontal Gene Transfer in Human-associated Microorganisms Inferred by Phylogenetic Reconstruction and Reconciliation. Sci. Rep..

[38] Smillie C.S., Smith M.B., Friedman J., Cordero O.X., David L.A., Alm E.J. (2011). Ecology Drives a Global Network of Gene Exchange Connecting the Human Microbiome. Nature.

[39] Davies M.G., Shera J., Van Domselaar G.H., Sriprakash K.S., McMillan D.J. (2009). A Novel Integrative Conjugative Element Mediates Genetic Transfer from Group G Streptococcus to Other β-Hemolytic Streptococci. J. Bacteriol..

[40] Brenciani A., Tiberi E., Bacciaglia A., Petrelli D., Varaldo P.E., Giovanetti E. (2011). Two Distinct Genetic Elements Are Responsible Forerm(TR)-Mediated Erythromycin Resistance in Tetracycline-Susceptible and Tetracycline-Resistant Strains of Streptococcus Pyogenes. Antimicrob. Agents Chemother..

[41] Wang H., Zhuang H., Ji S., Sun L., Zhao F., Wu D., Shen P., Jiang Y., Yu Y., Chen Y. (2021). Distribution of Erm Genes among MRSA Isolates with Resistance to Clindamycin in a Chinese Teaching Hospital. Infect. Genet. Evol..

[42] Giovanetti E. (2002). Conjugative Transfer of the Erm(A) Gene from Erythromycin-Resistant Streptococcus Pyogenes to Macrolide-Susceptible, S. Pyogenes, Enterococcus Faecalis and Listeria Innocua. J. Antimicrob. Chemother..

[43] Wolfe A.J., Toh E., Shibata N., Rong R., Kenton K., FitzGerald M., Mueller E.R., Schreckenberger P., Dong Q., Nelson D.E. (2012). Evidence of Uncultivated Bacteria in the Adult Female Bladder. J. Clin. Microbiol..

[44] Hilt E.E., McKinley K., Pearce M.M., Rosenfeld A.B., Zilliox M.J., Mueller E.R., Brubaker L., Gai X., Wolfe A.J., Schreckenberger P.C. (2013). Urine Is Not Sterile: Use of Enhanced Urine Culture Techniques to Detect Resident Bacterial Flora in the Adult Female Bladder. J. Clin. Microbiol..

[45] Vollstedt A., Baunoch D., Wolfe A., Luke N., Wojno K.J., Cline K., Belkoff L., Milbank A., Sherman N., Haverkorn R. (2020). Bacterial Interactions as Detected by Pooled Antibiotic Susceptibility Testing (P-AST) in Polymicrobial Urine Specimens. J. Surg. Urol..

[46] Zhou X., Cuasquer G.J.P., Li Z., Mang H.P., Lv Y. (2021). Occurrence of Typical Antibiotics, Representative Antibiotic-resistant Bacteria, and Genes in Fresh and Stored Source-separated Human Urine. Environ. Int..

[47] Wistrand-Yuen E., Knopp M., Hjort K., Koskiniemi S., Berg O.G., Andersson D.I. (2018). Evolution of High-level Resistance during Low-level Antibiotic Exposure. Nat. Commun..

[48] Ding M., Ye Z., Liu L., Wang W., Chen Q., Zhang F., Wang Y., Sjöling Å., Martín-Rodríguez A.J., Hu R. (2022). Subinhibitory Antibiotic Concentrations Promote the Horizontal Transfer of Plasmid-borne Resistance Genes from *Klebsiellae pneumoniae* to *Escherichia coli*. Front. Microbiol..

[49] Morley V.J., Woods R.J., Read A.F. (2019). Bystander Selection for Antimicrobial Resistance: Implications for Patient Health. Trends Microbiol..

[50] Chua K.P., Fischer M.A., Rahman M., Linder J.A. (2024). Changes in the Appropriateness of US Outpatient Antibiotic Prescribing After the Coronavirus Disease 2019 Outbreak: An Interrupted Time Series Analysis of 2016-2021 Data. Clin. Infect. Dis..

[51] Da Cunha V., Davies M.R., Douarre P.-E., Rosinski-Chupin I., Margarit I., Spinali S., Perkins T., Lechat P., Dmytruk N., Sauvage E. (2014). Streptococcus agalactiae Clones Infecting Humans Were Selected and Fixed through the Extensive Use of Tetracycline. Nat. Commun..

[52] Alcock B.P., Raphenya A.R., Lau T.T.Y., Tsang K.K., Bouchard M., Edalatmand A., Huynh W., Nguyen A.-L.V., Cheng A.A., Liu S. (2020). CARD 2020: Antibiotic Resistome Surveillance with the Comprehensive Antibiotic Resistance Database. Nucleic Acids Res..

[53] Antipov D., Korobeynikov A., McLean J.S., Pevzner P.A. (2015). HybridSPAdes: An Algorithm for Hybrid Assembly of Short and Long Reads. Bioinformatics.

[54] Lin Y., Yuan J., Kolmogorov M., Shen M.W., Chaisson M., Pevzner P.A. (2016). Assembly of Long Error-Prone Reads Using de Bruijn Graphs. Proc. Natl. Acad. Sci. USA.

[55] Koren S., Walenz B.P., Berlin K., Miller J.R., Bergman N.H., Phillippy A.M. (2017). Canu: Scalable and Accurate Long-Read Assembly via Adaptivek-Mer Weighting and Repeat Separation. Genome Res..

[56] Walker B.J., Abeel T., Shea T., Priest M., Abouelliel A., Sakthikumar S., Cuomo C.A., Zeng Q., Wortman J., Young S.K. (2014). Pilon: An Integrated Tool for Comprehensive Microbial Variant Detection and Genome Assembly Improvement. PLoS ONE.

[57] Gurevich A., Saveliev V., Vyahhi N., Tesler G. (2013). QUAST: Quality Assessment Tool for Genome Assemblies. Bioinformatics.

[58] Hunt M., Silva N.D., Otto T.D., Parkhill J., Keane J.A., Harris S.R. (2015). Circlator: Automated Circularization of Genome Assemblies Using Long Sequencing Reads. Genome Biol..

[59] Tatusova T., Di Cuccio M., Badretdin A., Chetvernin V., Nawrocki E.P., Zaslavsky L., Lomsadze A., Pruitt K.D., Borodovsky M., Ostell J. (2016). NCBI Prokaryotic Genome Annotation Pipeline. Nucleic Acids Res..

[60] Lao J., Guédon G., Lacroix T., Charron-Bourgoin F., Libante V., Loux V., Chiapello H., Payot S., Leblond-Bourget N. (2020). Abundance, Diversity and Role of ICEs and IMEs in the Adaptation of Streptococcus salivarius to the Environment. Genes.

[61] Ambroset C., Coluzzi C., Guédon G., Devignes M., Loux V., Lacroix T., Payot S., Leblond-Bourget N. (2016). New Insights into the Classification and Integration Specificity of Streptococcus Integrative Conjugative Elements through Extensive Genome Exploration. Front. Microbiol..

[62] Wang M., Goh Y.-X., Tai C., Wang H., Deng Z., Ou H.-Y. (2022). VRprofile2: Detection of Antibiotic Resistance-Associated Mobilome in Bacterial Pathogens. Nucleic Acids Res..

[63] Sullivan M.J., Petty N.K., Beatson S.A. (2011). Easyfig: A Genome Comparison Visualizer. Bioinformatics.

