# Supplementary material for: Macrolide Resistance in the Aerococcus urinae Complex: Implications for Integrative and Conjugative Elements
Source: Antibiotics (Basel). 2024 May 10;13(5):433. doi: 10.3390/antibiotics13050433 (PMC11117264; doi:10.3390/antibiotics13050433)
Supplement: Supplementary file 1 [file antibiotics-13-00433-s001.zip › antibiotics-2986726-supplementary.pdf]

## Supplementary Materials

**Table S1. List of Isolates**

| Number | Isolation Year | Collection Method | Growth at 10 μg/ml Azithromycin | Growth at 100 μg/ml Azithromycin | *ermA* |
|---|---|---|---|---|---|
| 1 | 2017 | void | 1 | 0 | 0 |
| 2 | 2017 | cath | 1 | 0 | 0 |
| 3 | 2017 | cath | 1 | 1 | 1 |
| 4 | 2017 | cath | 1 | 0 | 0 |
| 5 | 2017 | void | 1 | 1 | 1 |
| 6 | 2017 | void | 1 | 1 | 1 |
| 7 | 2017 | pswab | 1 | 0 | 0 |
| 8 | 2017 | vswab | 1 | 0 | 0 |
| 9 | 2017 | cath | 1 | 1 | 1 |
| 10 | 2017 | vswab | 1 | 1 | 1 |
| 11 | 2017 | pswab | 1 | 1 | 1 |
| 12 | 2017 | void | 1 | 1 | 1 |
| 13 | 2017 | vswab | 1 | 0 | 0 |
| 14 | 2017 | pswab | 1 | 0 | 0 |
| 15 | 2017 | void | 1 | 0 | 0 |
| 16 | 2018 | void | 1 | 0 | 0 |
| 17 | 2018 | vswab | 1 | 1 | 1 |
| 18 | 2018 | void | 1 | 0 | 0 |
| 19 | 2018 | cath | 1 | 1 | 1 |
| 20 | 2018 | cath | 1 | 0 | 0 |
| 21 | 2018 | cath | 1 | 1 | 1 |
| 22 | 2018 | void | 1 | 0 | 0 |
| 23 | 2018 | cath | 1 | 0 | 0 |
| 24 | 2018 | vswab | 1 | 0 | 0 |
| 25 | 2018 | pswab | 1 | 0 | 0 |
| 26 | 2018 | void | 1 | 1 | 1 |
| 27 | 2018 | void | 1 | 1 | 1 |
| 28 | 2018 | void | 1 | 0 | 1 |
| 29 | 2018 | cath | 1 | 0 | 0 |
| 30 | 2018 | cath | 1 | 0 | 0 |
| 31 | 2018 | vswab | 0 | 0 | 0 |
| 32 | 2018 | cath | 1 | 1 | 1 |
| 33 | 2018 | cath | 1 | 1 | 1 |
| 34 | 2018 | cath | 1 | 0 | 0 |
| 35 | 2018 | void | 0 | 0 | 0 |
| 36 | 2018 | cath | 1 | 0 | 0 |
| 37 | 2018 | pswab | 1 | 0 | 0 |
| 38 | 2018 | void | 0 | 0 | 0 |
| 39 | 2018 | vswab | 1 | 0 | 0 |
| 40 | 2018 | cath | 1 | 0 | 0 |

| | | | | | |
|---|---|---|---|---|---|
| 41 | 2018 | void | 1 | 0 | 0 |
| 42 | 2018 | void | 1 | 0 | 0 |
| 43 | 2018 | vswab | 0 | 0 | 0 |
| 44 | 2018 | pswab | 0 | 0 | 0 |
| 45 | 2018 | upper | 1 | 0 | 0 |
| 46 | 2018 | vswab | 1 | 0 | 0 |
| 47 | 2018 | cath | 1 | 1 | 1 |
| 48 | 2018 | cath | 0 | 0 | 0 |
| 49 | 2018 | void | 0 | 0 | 0 |
| 50 | 2018 | void | 0 | 0 | 0 |
| 51 | 2018 | vswab | 0 | 0 | 0 |
| 52 | 2018 | void | 1 | 0 | 0 |
| 53 | 2018 | cath | 0 | 0 | 0 |
| 54 | 2018 | void | 1 | 0 | 0 |
| 55 | 2018 | pswab | 0 | 0 | 0 |
| 56 | 2018 | void | 1 | 1 | 1 |
| 57 | 2018 | cath | 1 | 1 | 1 |
| 58 | 2018 | cath | 1 | 1 | 1 |
| 59 | 2018 | cath | 1 | 1 | 1 |
| 60 | 2018 | pswab | 0 | 0 | 0 |
| 61 | 2018 | pswab | 1 | 0 | 0 |
| 62 | 2018 | cath | 0 | 0 | 0 |
| 63 | 2018 | vswab | 1 | 1 | 1 |
| 64 | 2018 | void | 1 | 1 | 1 |
| 65 | 2018 | pswab | 1 | 1 | 1 |
| 66 | 2018 | cath | 1 | 1 | 1 |
| 67 | 2018 | vswab | 0 | 0 | 0 |
| 68 | 2018 | pswab | 0 | 0 | 0 |
| 69 | 2018 | void | 0 | 0 | 0 |
| 70 | 2018 | vswab | 1 | 1 | 1 |
| 71 | 2019 | void | 1 | 0 | 0 |
| 72 | 2019 | cath | 1 | 1 | 0 |
| 73 | 2019 | vswab | 1 | 1 | 0 |
| 74 | 2019 | cath | 0 | 0 | 0 |
| 75 | 2019 | cath | 0 | 0 | 0 |
| 76 | 2019 | cath | 0 | 0 | 0 |
| 77 | 2019 | cath | 0 | 0 | 0 |
| 78 | 2020 | void | 0 | 0 | 0 |
| 79 | 2020 | cath | 1 | 1 | 1 |
| 80 | 2020 | cath | 1 | 1 | 1 |
| 81 | 2020 | void | 1 | 1 | 1 |
| 82 | 2020 | cath | 1 | 1 | 1 |
| 83 | 2020 | void | 0 | 0 | 0 |
| 84 | 2020 | cath | 0 | 0 | 0 |
| 85 | 2021 | cath | 1 | 1 | 1 |
| 86 | 2021 | void | 1 | 1 | 1 |

| | | | | | |
|---|---|---|---|---|---|
| 87 | 2021 | cath | 1 | 1 | 1 |
| 88 | 2021 | void | 0 | 0 | 0 |
| 89 | 2021 | cath | 1 | 1 | 1 |
| 90 | 2021 | cath | 1 | 1 | 1 |
| 91 | 2021 | cath | 1 | 0 | 0 |
| 92 | 2021 | void | 1 | 1 | 1 |
| 93 | 2021 | cath | 0 | 0 | 1 |
| 94 | 2021 | void | 1 | 1 | 1 |
| 95 | 2021 | cath | 1 | 0 | 0 |
| 96 | 2021 | cath | 0 | 0 | 0 |
| 97 | 2021 | cath | 0 | 0 | 1 |
| 98 | 2021 | cath | 0 | 0 | 0 |
| 99 | 2021 | cath | 1 | 1 | 1 |
| 100 | 2021 | cath | 1 | 0 | 0 |
| 101 | 2021 | cath | 1 | 0 | 0 |
| 102 | 2021 | void | 1 | 1 | 1 |
| 103 | 2021 | cath | 1 | 1 | 1 |
| 104 | 2021 | cath | 1 | 0 | 0 |
| 105 | 2021 | cath | 1 | 0 | 0 |
| 106 | 2021 | cath | 0 | 0 | 0 |
| 107 | 2021 | cath | 1 | 1 | 1 |
| 108 | 2021 | cath | 1 | 1 | 1 |
| 109 | 2021 | cath | 1 | 0 | 0 |
| 110 | 2021 | void | 0 | 0 | 1 |
| 111 | 2021 | stone | 1 | 1 | 1 |
| 112 | 2021 | cath | 1 | 0 | 0 |
| 113 | 2021 | cath | 1 | 1 | 1 |
| 114 | 2021 | cath | 1 | 0 | 0 |
| 115 | 2021 | cath | 0 | 0 | 0 |
| 116 | 2021 | void | 0 | 0 | 0 |
| 117 | 2021 | cath | 1 | 0 | 0 |
| 118 | 2021 | cath | 1 | 0 | 0 |
| 119 | 2021 | cath | 1 | 0 | 0 |
| 120 | 2021 | void | 1 | 1 | 1 |
| 121 | 2021 | cath | 0 | 0 | 0 |
| 122 | 2021 | cath | 1 | 0 | 0 |
| 123 | 2021 | cath | 1 | 0 | 0 |
| 124 | 2021 | cath | 1 | 0 | 0 |
| 125 | 2022 | cath | 1 | 0 | 0 |
| 126 | 2022 | cath | 1 | 0 | 0 |
| 127 | 2022 | void | 1 | 1 | 1 |
| 128 | 2022 | cath | 1 | 0 | 0 |
| 129 | 2022 | void | 1 | 1 | 1 |
| 130 | 2022 | cath | 1 | 1 | 1 |
| 131 | 2022 | cath | 1 | 1 | 1 |
| 132 | 2022 | cath | 1 | 1 | 1 |

| | | | | | |
|---|---|---|---|---|---|
| 133 | 2022 | cath | 0 | 0 | 0 |
| 134 | 2022 | void | 1 | 1 | 1 |
| 135 | 2022 | cath | 1 | 0 | 1 |
| 136 | 2022 | cath | 1 | 1 | 1 |
| 137 | 2022 | cath | 1 | 1 | 1 |
| 138 | 2022 | cath | 1 | 0 | 0 |
| 139 | 2022 | cath | 1 | 1 | 1 |
| 140 | 2022 | void | 1 | 0 | 0 |
| 141 | 2022 | vswab | 1 | 0 | 0 |
| 142 | 2022 | cath | 1 | 1 | 1 |
| 143 | 2023 | cath | 1 | 0 | 0 |
| 144 | 2023 | cath | 1 | 0 | 0 |
| 145 | 2023 | uswab | 1 | 1 | 1 |
| 146 | 2023 | puswab | 1 | 0 | 0 |
| 147 | 2023 | CTIP | 1 | 1 | 1 |
| 148 | 2023 | cath | 1 | 1 | 1 |
| 149 | 2023 | cath | 1 | 1 | 1 |
| 150 | 2023 | vswab | 1 | 1 | 1 |
| 151 | 2023 | puswab | 1 | 0 | 0 |
| 152 | 2023 | uswab | 1 | 1 | 1 |
| 153 | 2023 | cath | 1 | 1 | 1 |
| 154 | 2023 | vswab | 1 | 1 | 1 |
| 155 | 2023 | void | 0 | 0 | 0 |
| 156 | 2023 | CTIP | 1 | 0 | 0 |
| 157 | 2023 | void | 0 | 0 | 0 |
| 158 | 2023 | void | 0 | 0 | 0 |
| 159 | 2023 | void | 0 | 0 | 0 |
| 160 | 2023 | cath | 1 | 1 | 1 |
| 161 | 2023 | void | 1 | 1 | 1 |
| 162 | 2023 | puswab | 1 | 1 | 1 |
| 163 | 2023 | cath | 1 | 1 | 1 |
| 164 | 2023 | void | 1 | 1 | 1 |
| 165 | 2023 | puswab | 0 | 0 | 0 |
| 166 | 2023 | vswab | 1 | 1 | 1 |
| 167 | 2023 | void | 1 | 1 | 1 |
| 168 | 2023 | void | 1 | 1 | 1 |
| 169 | 2023 | vswab | 1 | 1 | 1 |
| 170 | 2023 | cath | 1 | 1 | 1 |
| 171 | 2023 | void | 1 | 1 | 1 |
| 172 | 2023 | vswab | 1 | 0 | 0 |
| 173 | 2023 | vswab | 1 | 1 | 1 |
| 174 | 2023 | void | 1 | 1 | 1 |
| 175 | 2023 | cath | 1 | 1 | 1 |
| 176 | 2023 | vswab | 1 | 1 | 1 |
| 177 | 2023 | puswab | 1 | 1 | 1 |
| 178 | 2023 | cath | 1 | 1 | 1 |

| | | | | | |
|---|---|---|---|---|---|
| 179 | 2023 | vswab | 1 | 1 | 1 |
| 180 | 2023 | cath | 1 | 0 | 0 |
| 181 | 2023 | puswab | 1 | 0 | 0 |
| 182 | 2023 | vswab | 1 | 1 | 1 |
| 183 | 2023 | vswab | 1 | 0 | 0 |
| 184 | 2023 | cath | 1 | 0 | 0 |
| 185 | 2023 | cath | 1 | 1 | 1 |
| 186 | 2023 | cath | 1 | 0 | 0 |
| 187 | 2023 | void | 1 | 1 | 1 |
| 188 | 2023 | void | 1 | 1 | 1 |
| 189 | 2023 | void | 1 | 0 | 1 |

pswab = perineal swab, vswab = vaginal swab, uswab = urethral swab, CTIP = catheter tip, cath = transurethral catheter urine, void = voided urine, puswab = periurethral swab, stone = kidney stone. 1= positive result 0= negative result

**Table S2. List of Analyzed Genomes**

| Strain Name | Accession No. | BioProject |
|---|---|---|
| 151250009-4-258-51 | GCF_014050445.1 | PRJNA649272 |
| 151250015-1-258-55 | GCF_014050435.1 | PRJNA649272 |
| 151250015-2-258-56 | GCF_014050425.1 | PRJNA649272 |
| 1KP-2016 | GCF_002252085.1 | PRJNA383933 |
| ACS-120-V-Col10a | GCF_000193205.1 | PRJNA51073 |
| Aerococcus_suis_DSM_21500 | GCF_900176325.1 | PRJEB20326 |
| As-24-U13 | GCF_905109795.1 | PRJEB36767 |
| As-25-U14 | GCF_905125215.1 | PRJEB36767 |
| As-29b-U14 | GCF_905113005.1 | PRJEB36767 |
| As-34-B09 | GCF_905115175.1 | PRJEB36767 |
| As-41-B14 | GCF_905113015.1 | PRJEB36767 |
| As-46-U14 | GCF_905113025.1 | PRJEB36767 |
| As-55-B15 | GCF_905113035.1 | PRJEB36767 |
| As-56-U15 | GCF_905109855.1 | PRJEB36767 |
| ATCC_11563_=_CCUG_4311 | GCF_000178435.1 | PRJNA308559 |
| ATCC_51268 | GCF_002087935.1 | PRJNA379934 |
| Au-01-U13 | GCF_905113045.1 | PRJEB36767 |
| Au-02-B96 | GCF_905115225.1 | PRJEB36767 |
| Au-03-U96 | GCF_905113065.1 | PRJEB36767 |
| Au-04-B04 | GCF_905113075.1 | PRJEB36767 |
| Au-06-U13 | GCF_905113055.1 | PRJEB36767 |
| Au-07-B93 | GCF_905111135.1 | PRJEB36767 |
| Au-08-B04 | GCF_905125195.1 | PRJEB36767 |
| Au-09-B94 | GCF_905115135.1 | PRJEB36767 |
| Au-10-B10 | GCF_905125205.1 | PRJEB36767 |
| Au-11-U84 | GCF_905125305.1 | PRJEB36767 |
| Au-12-B98 | GCF_905125255.1 | PRJEB36767 |
| Au-13-B13 | GCF_905111125.1 | PRJEB36767 |
| Au-15-B94 | GCF_905125225.1 | PRJEB36767 |

| | | |
|---|---|---|
| Au-16-B92 | GCF_905115205.1 | PRJEB36767 |
| Au-17-B94 | GCF_905125275.1 | PRJEB36767 |
| Au-18-B93 | GCF_905125265.1 | PRJEB36767 |
| Au-19-H93 | GCF_905125335.1 | PRJEB36767 |
| Au-20-U84 | GCF_905111885.1 | PRJEB36767 |
| Au-21-B87 | GCF_905125295.1 | PRJEB36767 |
| Au-23-B94 | GCF_905115095.1 | PRJEB36767 |
| Au-26-U84 | GCF_905125245.1 | PRJEB36767 |
| Au-28-U13 | GCF_905125235.1 | PRJEB36767 |
| Au-29a-U14 | GCF_905125315.1 | PRJEB36767 |
| AU3 | GCF_001649715.1 | PRJNA315093 |
| Au-30-B02 | GCF_905111875.1 | PRJEB36767 |
| Au-31-B94 | GCF_905125325.1 | PRJEB36767 |
| Au-33-U84 | GCF_905115125.1 | PRJEB36767 |
| Au-43-B13 | GCF_905112965.1 | PRJEB36767 |
| Au-44-B14 | GCF_905125285.1 | PRJEB36767 |
| Au-45-U14 | GCF_905115075.1 | PRJEB36767 |
| Au-47-U14 | GCF_905112955.1 | PRJEB36767 |
| Au-49-B14 | GCF_905115085.1 | PRJEB36767 |
| Au-50-U14 | GCF_905115115.1 | PRJEB36767 |
| Au-51-B15 | GCF_905112975.1 | PRJEB36767 |
| Au-52-U15 | GCF_905115165.1 | PRJEB36767 |
| Au-53-B14 | GCF_905115105.1 | PRJEB36767 |
| Au-54-U14 | GCF_905112985.1 | PRJEB36767 |
| Au-57-B15 | GCF_905115155.1 | PRJEB36767 |
| Au-58-U15 | GCF_905115195.1 | PRJEB36767 |
| Au-59-B15 | GCF_905112995.1 | PRJEB36767 |
| Au-60-U15 | GCF_905115145.1 | PRJEB36767 |
| AV208 | GCF_001719605.1 | PRJNA339485 |
| CCUG28094 | GCF_001543205.1 | PRJNA308559 |
| CCUG28831 | GCF_001543105.1 | PRJNA308559 |
| CCUG42038B | GCF_001543245.1 | PRJNA308559 |
| CCUG43001 | GCF_001543145.1 | PRJNA308559 |
| CCUG4311 | GCF_001543285.1 | PRJNA308559 |
| CDC-1515-U85 | GCA_026694535.1 | PRJNA876651 |
| CDC-1656-U92 | GCA_026695435.1 | PRJNA876651 |
| CDC-1871-U94 | GCA_026695425.1 | PRJNA876651 |
| CDC-3352-U95 | GCA_026694515.1 | PRJNA876651 |
| CDC-944-U94 | GCA_026695485.1 | PRJNA876651 |
| DSM_15634 | GCF_900103385.1 | PRJEB16196 |
| DSM_15819_=_CCUG_28831 | GCF_001466745.1 | PRJNA175732 |
| DSM_20341_=_CCUG_28094 | GCF_000425085.1 | PRJNA308559 |
| FDAARGOS_249 | GCF_002083135.2 | PRJNA231221 |
| FDAARGOS_911 | GCF_016026975.1 | PRJNA231221 |
| HMSC035B07 | GCF_001836025.1 | PRJNA296312 |

| | | |
|---|---|---|
| HMSC061A03 | GCF_001813115.1 | PRJNA300046 |
| HMSC062A02 | GCF_001809535.1 | PRJNA296233 |
| HMSC062B07 | GCF_001809895.1 | PRJNA296287 |
| HMSC06H08 | GCF_001807425.1 | PRJNA269898 |
| HMSC072A12 | GCF_001811965.1 | PRJNA299983 |
| HMSC075D05 | GCF_001811135.1 | PRJNA299938 |
| HMSC10H05 | GCF_001806975.1 | PRJNA269853 |
| HMSC23C02 | GCF_001806805.1 | PRJNA269827 |
| KA00635 | GCF_001552755.1 | PRJNA272079 |
| LL1 | GCF_000262085.1 | PRJNA159617 |
| LUND-01-B14 | GCA_026695685.1 | PRJNA876651 |
| LUND-02-B14 | GCA_026695635.1 | PRJNA876651 |
| LUND-04-B14 | GCA_026695605.1 | PRJNA876651 |
| LUND-06-B14 | GCA_026694875.1 | PRJNA876651 |
| LUND-10-B14 | GCA_026694505.1 | PRJNA876651 |
| LUND-12-B13 | GCA_026695705.1 | PRJNA876651 |
| LUND-14-B13 | GCA_026695665.1 | PRJNA876651 |
| LUND-16-B13 | GCA_026694895.1 | PRJNA876651 |
| LUND-20-B13 | GCA_026695625.1 | PRJNA876651 |
| LUND-25-B13 | GCA_026695505.1 | PRJNA876651 |
| LUND-33-B12 | GCA_026695585.1 | PRJNA876651 |
| LUND-35-B12 | GCA_026695535.1 | PRJNA876651 |
| LUND-36-B12 | GCA_026694865.1 | PRJNA876651 |
| LUND-40-B12 | GCA_026694525.1 | PRJNA876651 |
| LUND-41-B12 | GCA_026695525.1 | PRJNA876651 |
| LUND-44-B12 | GCA_026695025.1 | PRJNA876651 |
| NBRC_15544_=_CCUG_36881<br>_NBRC_15544 | GCF_001544335.1 | PRJNA308559 |
| NCTC7595 | GCF_900445105.1 | PRJEB6403 |
| NCTC8251 | GCF_900445095.1 | PRJEB6403 |
| NLD-005-U95 | GCA_026694675.1 | PRJNA876651 |
| NLD-015-U95 | GCA_026694665.1 | PRJNA876651 |
| NLD-022-U95 | GCA_026694725.1 | PRJNA876651 |
| NLD-036-U95 | GCA_026694685.1 | PRJNA876651 |
| NLD-049-U95 | GCA_026694645.1 | PRJNA876651 |
| NLD-059-U95 | GCA_026695445.1 | PRJNA876651 |
| NLD-060-U95 | GCA_026694965.1 | PRJNA876651 |
| NLD-066-U95 | GCA_026695565.1 | PRJNA876651 |
| NLD-971-U95 | GCA_026694485.1 | PRJNA876651 |
| SJQ22 | GCF_003797145.1 | PRJNA504512 |
| SLA-40126-U13 | GCA_026695825.1 | PRJNA876651 |
| SLA-43350-U13 | GCA_026695805.1 | PRJNA876651 |
| SLA-43565-U13 | GCA_026695785.1 | PRJNA876651 |
| SLA-45893-U13 | GCA_026695765.1 | PRJNA876651 |
| SLA-48199-U13 | GCA_026695745.1 | PRJNA876651 |
| SLA-48243-U13 | GCA_026694815.1 | PRJNA876651 |

| | | |
|---|---|---|
| SLA-48590-U13 | GCA_026695725.1 | PRJNA876651 |
| T43 | GCF_014931015.1 | PRJNA668452 |
| UMB0071 | GCF_003286525.1 | PRJNA316969 |
| UMB0072 | GCF_002847665.1 | PRJNA316969 |
| UMB0072b | GCF_002884575.1 | PRJNA316969 |
| UMB0080 | GCF_002871915.2 | PRJNA316969 |
| UMB0088 | GCF_002884955.2 | PRJNA316969 |
| UMB0126 | GCF_002847705.1 | PRJNA316969 |
| UMB0139 | GCF_002847725.1 | PRJNA316969 |
| UMB0232 | GCF_002847625.1 | PRJNA316969 |
| UMB0239 | GCF_003286895.1 | PRJNA316969 |
| UMB0240 | GCF_002871935.1 | PRJNA316969 |
| UMB0267 | GCF_003286875.3 | PRJNA316969 |
| UMB0337 | GCF_003286845.2 | PRJNA316969 |
| UMB0509 | GCF_003286825.2 | PRJNA316969 |
| UMB0553 | GCF_003286805.1 | PRJNA316969 |
| UMB0621 | GCF_003286515.1 | PRJNA316969 |
| UMB0637 | GCF_008726885.1 | PRJNA316969 |
| UMB0722 | GCF_003286755.1 | PRJNA316969 |
| UMB0844 | GCF_002861505.1 | PRJNA316969 |
| UMB0970 | GCF_008726845.1 | PRJNA316969 |
| UMB1016 | GCF_003286735.3 | PRJNA316969 |
| UMB1741 | GCF_003286715.1 | PRJNA316969 |
| UMB2126 | GCF_008726675.1 | PRJNA316969 |
| UMB2325 | GCF_003286725.1 | PRJNA316969 |
| UMB2354 | GCF_003286695.1 | PRJNA316969 |
| UMB2879 | GCF_003286665.1 | PRJNA316969 |
| UMB3440 | GCF_003286595.3 | PRJNA316969 |
| UMB3669 | GCF_003286645.3 | PRJNA316969 |
| UMB5628 | GCF_003286585.1 | PRJNA316969 |
| UMB623 | GCF_008726925.1 | PRJNA316969 |
| UMB6497 | GCF_003286635.1 | PRJNA316969 |
| UMB7049 | GCF_008726475.2 | PRJNA316969 |
| UMB7382 | GCF_003286565.1 | PRJNA316969 |
| UMB7480 | GCF_003286555.1 | PRJNA316969 |
| UMB8614 | GCF_008726385.1 | PRJNA316969 |
| UMB8662 | GCF_008726315.1 | PRJNA316969 |
| UMB8711 | GCF_008726285.1 | PRJNA316969 |
| USDA-ARS-USMARC-56713 | GCF_001518795.1 | PRJNA281531 |

**Table S3. NCBI Deposit of Complete Genomes**

| | Illumina Raw Reads | Oxford Nanopore Raw Reads | Hybrid Assembly |
|---|---|---|---|
| UMB3669 | SRX3914940 | SRX20822257 | GCA_003286645.3 |
| UMB0337 | SRX20822236 | SRX20822241 | GCA_003286845.2 |
| UMB7049 | SRX6878814 | SRX20822261 | GCA_008726475.2 |
| UMB0080 | SRX3104357 | SRX20822271 | GCA_002871915.2 |
| UMB0509 | SRX3914955 | SRX20822243 | GCA_003286825.2 |
| UMB0088 | SRX3104358 | SRX20822238 | GCA_002884955.2 |
| UMB3440 | SRX3914941 | SRX20822256 | GCA_003286595.3 |
| UMB1016 | SRX3914949 | SRX20822252 | GCA_003286735.2 |

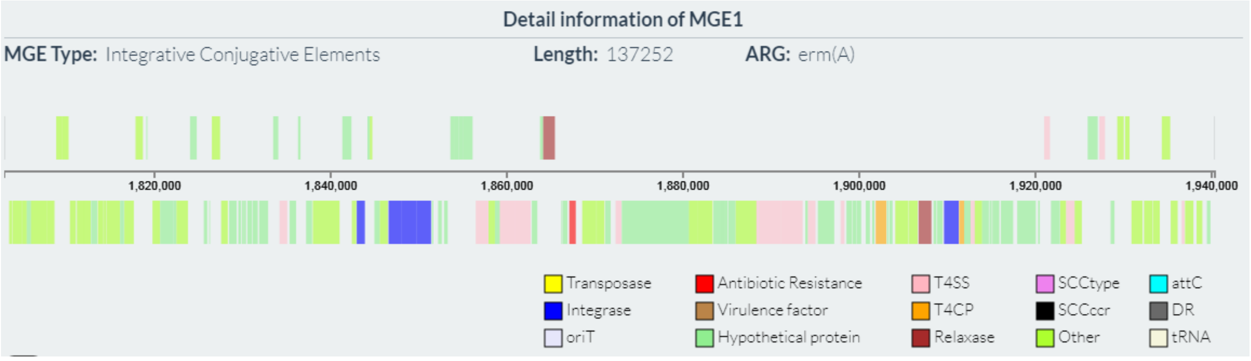


**Figure S1 – VRprofile2 output of ICEAloyUMB0088**

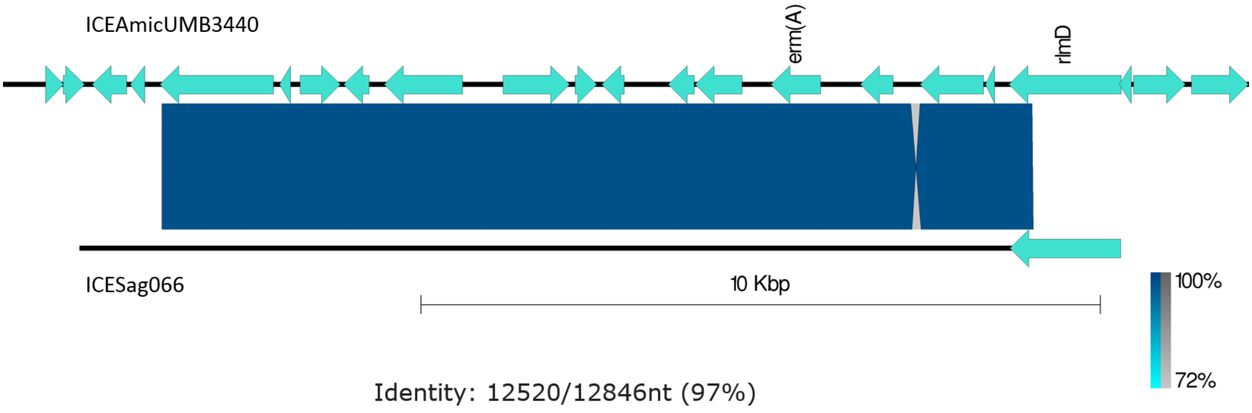


**Figure S2 – Nucleotide homology of AUC ICEAmicUMB3440 and *Streptococcus agalactiae* ICESag066**
